# Shaping the future of healthcare: improving quality and safety through integrating simulation into Public Health education

**DOI:** 10.3389/fpubh.2024.1446708

**Published:** 2024-08-12

**Authors:** Tatjana Baldovin, Francesco Bassan, Chiara Bertoncello, Alessandra Buja, Silvia Cocchio, Marco Fonzo, Vincenzo Baldo

**Affiliations:** Hygiene and Public Health Unit, Department of Cardiac, Thoracic, Vascular Sciences and Public Health, School of Specialization in Hygiene and Preventive Medicine, University of Padua, Padua, Italy

**Keywords:** simulation-based education, innovative education and pedagogy/teaching methods, patient safety, Quality Improvement, Public Health education and health promotion

## Abstract

This perspective focuses on the role of healthcare simulation in training and implementing processes aimed at improving the quality of care and patient safety. Evidence of the effectiveness of simulation in improving clinical performance, reducing healthcare costs and raising professional education standards is presented. In light of this evidence, we propose to consider simulation-based education as an integrative training modality in the preparation of health professionals in the field of Public Health. A pilot project is presented with the aim of training professionals capable of further contributing to improving the quality and safety of patients through an interdisciplinary and innovative approach.

## Introduction

Healthcare simulation is expanding rapidly (1) all over the world including low- and middle-income countries. In these regions, the use of simulation-based programs has proven promising beyond their demonstrated educational objectives even in the perspective of addressing the United Nations Sustainable Development Goals, such as good health and wellbeing and quality education (2, 3).

Rephrasing the definition of Gaba, simulation is more than a technological tool; it is a distinct technique aiming to replace or enhance real experiences through guided, immersive scenarios replicating substantial aspects of the real world (4). As a methodology, it adheres to precise definitions and regulations. The Italian Ministry of Health acknowledged its significance, issuing the “Linee di indirizzo sullo sviluppo della simulazione in sanità in Italia” in 2022, providing guidelines on quality standards for programs using simulation as a core teaching value (5).

While simulation's establishment in professional education is recognized, its role as a tool for healthcare Quality Improvement (QI) and Patient Safety (PS) presents exciting challenges (6). A study showed how a simulation program was able to improve door-to-needle times and patient outcomes in acute ischemic stroke (7). Furthermore, other studies highlighted the positive impact of simulation on QI, as well as on the possibility of financial savings from a sustainability perspective (8, 9). These emerging findings present interesting insights for potential future horizons in Public Health.

Brazil introduced the term “translational simulation” into the literature (10). It is an invitation to consider simulation from a functional perspective, focusing on the purpose of improving quality and safety, rather than other modalities or delivery processes (e.g., high/low fidelity, *in situ*, and standardized patients). From the perspective of purpose, simulation activities can be considered either diagnostic (to identify problems) or interventional (to identify solutions to problems), or both. The focus is notably directed toward addressing systemic issues (e.g., exploring work environments and/or the people within them, targeting interventions focused on clinical performance/patient outcomes, designing and testing planned infrastructure or interventions), thereby contributing to safety, performance issues, and ultimately to QI (11).

There is an opportunity to integrate simulation into traditional study models of QI, not as an alternative, but rather as a complementary tool, bringing benefits. A study proved that incorporating simulation into the healthcare failure mode and effects analysis (FMEA) framework has allowed for the gathering of additional information for a more comprehensive analysis (12).

Generally, clinicians have limited knowledge of practices related to QI, and conversely, those involved in QI often exhibit a poor awareness of the potential of simulation. This dissonance is likely also attributable to the fact that these two disciplines have historical roots in different conceptual grounds. Therefore, we agree with what Brazil and colleagues argue: “Skills development is likely to be enhanced if simulation and QI staff in the same institutions have opportunities to work closely together” (13).

With this proposal, it is hoped to provide healthcare professionals involved in improving PS and quality, as well as frontline clinicians, an innovative opportunity to mutually benefit from each other's expertise.

## Integration of simulation into the curriculum of Public Health School. A pilot project

The integration of simulation into the School of Public Health curriculum for undergraduate and postgraduate students is a pioneering and innovative proposal inspired by the World Health Organization (WHO) “Global Plan of Action for Patient Safety 2021–2030” (14) and by “Global Consensus statement on simulation-based practice in healthcare” (15). The WHO and the Global Consensus aim to improve the safety and quality of healthcare through a series of strategic objectives and actions, including the implementation of innovative methodologies such as simulation.

Additionally, the Italian Ministry of Health's publication of the national strategic document “Linee di indirizzo sullo sviluppo della simulazione in sanità in Italia” (5) in 2022 provides a technical guidance useful to align this proposal with national and international standards.

The pilot project, defined as the “Padua Public Health Simulation Lab” (PPHSL) has two objectives:

1. To use simulation as an integrative tool for Public Health courses provided by the University;
2. To train Public Health professionals as experts in simulation methodology.

### Simulation as an integrative tool for Public Health training. Why?

The integration of the educational curriculum with simulation offers trainees the valuable opportunity to apply themselves in safe contexts. By engaging in simulated scenarios that closely mirror real-life situations, participants can both improve technical skills in hygiene, preventive medicine and Public Health, and develop crucial non-technical skills such as leadership, decision-making, teamwork, situational awareness and communication.

### Training Public Health professionals in simulation. Why?

Throughout their professional careers, Public Health specialists may be called upon to fill a wide range of roles up to the top and managerial levels.

Whatever the job position held, inter-professional and interdisciplinary collaboration are fundamental components of the work environment, as are technical and non-technical skills. This occurs both locally, for example when involved in managerial and QI tasks of healthcare services, and in international contexts.

Training Public Health specialists in mastering simulation methodology means providing them with an additional tool that may prove valuable in contributing to several strategic objectives identified by the WHO Action Plan: ensuring the safety of every clinical process, building high-reliability health systems, designing and delivering safer care systems.

For example, a Public Health professional can acquire the important tool of debriefing. Debriefing is a well-structured tool in simulation-based education of guided analysis of an event with the aim of positively influencing future practice. In contrast, clinical debriefing (CD) is an emerging practice in the clinical setting with potential benefits for both staff and patients (16). A study shows significant improvement in neurological outcomes and a trend toward increased post-cardiac arrest survival following implementation of an interdisciplinary debriefing program (17). The expertise gained by the Public Health specialist in the field of simulation could be a facilitating factor in the implementation of CD in clinical practice.

In general, simulation skills can be valuable for the Public Health professional in proposing and supporting translational simulation projects (11). These projects are designed both to diagnose problems and to solve them through simulation-based interventions. The overall objective is to optimize the benefits for healthcare professionals, clinical teams, healthcare systems and patients.

### An overview of the program. How and what?

The PPHSL program (Table 1) has been developed through consultations among Public Health trainees, residents, academics and healthcare professionals. The program may appear ambitious, but the intention was to construct a progressive and adaptable roadmap deriving from a shared unique vision, embodied a synthesis of educational needs and the desire for educational innovation to address QI and PS.

**Table 1:** A synoptic overview of the proposed program for the Padua Public Health Simulation Lab.

**Program at a glance**				
**Padua Public Health Simulation Lab**				
**Sessions/General topic**		**Specific topic**	**Methods**	**Target**
I	Patient safety Risk management Quality Improvement	Hand hygiene Healthcare associated infection Infection prevention and control Personal protective equipment	Virtual reality sim Low-high fidelity sim Skill lab	Undergraduate Postgraduate Healthcare workers
II	Vaccine	Vaccine hesitancy	Virtual reality sim Standardized patient	Undergraduate Postgraduate Healthcare workers
III	Emergency in Public Health setting	Anaphylactic shock and other emergencies in vaccine clinic	Skill Lab (e.g., basic airway management, BLSD) High fidelity sim *in situ*	Postgraduate Healthcare workers
IV	Non-technical/soft skill for Public Health	Communication, leadership, decision making, situational awareness, and teamwork	Role play High fidelity sim	Postgraduate Healthcare workers
V	Preparedness	Disease outbreak Disaster medicine	From table top to full scale	Postgraduate Healthcare workers
VI	Train the trainer	Healthcare simulation methodology (fundamentals, scenario design and execution, and debriefing)	Skill lab, low-high fidelity, *in situ*, virtual reality, table top, and full scale	Postgraduate Healthcare workers

An inspiring guiding principle is the commitment to fostering a multidisciplinary and multi-professional safe-learning environment. Embracing a collaborative approach, the project aims to assemble teams comprising individuals from diverse disciplines and professional backgrounds. This approach not only mirrors the interdisciplinary nature of Public Health challenges but also enriches the learning experience by incorporating a variety of perspectives and expertise. It also provides an opportunity for sharing the ethical values of diversity, equity and inclusion.

The program adopts the SimZones model (18) and takes into account the WHO Patient safety curriculum guide (19) to effectively structure training sessions and facilitate skills development.

In each simulation exercise, participants will engage in a structured prebriefing and debriefing session. In addition, we will utilize the peer education approach to engage senior students in tutoring younger students.

The program will initially enlist the participation of a select group of medical students and Public Health residents. Furthermore, the curriculum will be tailored to the specific needs and learning goals of the participants, ensuring that educational activities are closely aligned with individual learning objectives.

The initial program includes simulation sessions based on the “Room of Horror” (20), conducted in both immersive virtual reality and high-fidelity formats. These sessions aim to introduce the topic of PS to undergraduate students and explore specific topics for postgraduates such as healthcare-associated infections and antimicrobial resistance.

A Skill Lab has been set up for undergraduate students, utilizing immersive virtual reality, to train them in hand hygiene.

Scenarios with standardized patients have been prepared to introduce the topic of vaccine hesitancy to undergraduate students (medical students, nurses, healthcare assistants) and to train postgraduates (Public Health residents) in enhancing both technical and non-technical skills. High-fidelity scenarios have been developed to train postgraduates in managing emergencies that may occur in a vaccination clinic, both rare but dangerous (such as anaphylactic shock) and more common but potentially critical to PS (such as syncope and trauma).

A progressive involvement of professionals interested in the train-the-trainer program, conducted by academics, is planned to support faculty development.

Planned evaluation will monitor student satisfaction and learning outcomes.

## Discussion

Listed below are the key aspects of simulation and the reasons why we believe simulation should be considered not only by clinicians but also by Public Health professionals and which constitute the rationale for our proposal.

- Education and Quality Improvement;
- Financial Saving;
- Training on Non-Technical Skills and Human Factors;
- Improvement of Clinical Outcomes;
- Patient and Caregivers Engagement;
- Innovation and research opportunities;
- Ethical Approach and Learning by doing in a safe environment.

### Application to education and Quality Improvement

Simulation has broad applications in both clinical educational and QI contexts. Andreatta's editorial highlights significant progress in resource-limited regions too, providing healthcare professionals with opportunities for education and clinical enhancement. This progress extends to diagnostic and testing domains, fostering improvements in healthcare quality through experimentation with new devices, techniques and process optimization (21).

Examples in the literature show simulation-based educational activities also to address specific Public Health issues such as epidemics, disaster medicine and prevention of Healthcare-Associated Infections (HAI) (9, 22–25). Ragazzoni et al. highlight the efficacy of virtual and hybrid simulation in training professionals for infection control, particularly in settings such as Ebola (26). Despite encouraging evidence in the context of preventing HAI, simulation is believed to be underused as it can effectively integrate into traditional curricula (27). Another study highlights the effectiveness of combining various simulation modalities in developing nursing students' skills in addressing vaccine hesitancy, a crucial issue for Public Health as one of the threats to global health (28, 29).

In the domain of QI, *in situ* simulation, conducted in actual patient care units, proves highly effective. Patterson et al. (30) identified 73 latent safety threats in the Emergency Department through 90 unannounced simulations, illustrating its potency. Other studies have emphasized its value in mitigating negative effects of hospital relocation and dealing with a Methicillin-Resistant Staphylococcus Aureus (MRSA) outbreak in a neonatal intensive care unit (31, 32). The recent SARS-CoV-2 pandemic has further highlighted the role of simulation in evaluating and improving preparedness and response in healthcare systems (33).

### Financial saving

Implementing simulation programs not only enhances safety and clinical practices but also produces tangible economic benefits. As an example, a substantial reduction in catheter-related infections in intensive care units, resulting in significant financial savings in hospital costs (9). In the surgical context, on the other hand, simulation-based training for residents reduced complications and costs associated with orthopedic procedures, demonstrating a positive return on investment (34).

A study on the identification and management of clinical deterioration in pediatric settings, indicated that regular simulation projects led to lasting improvements in PS and economic savings from reduced intensive care unit stays (35). Similarly, positive outcomes in care improvement and significant cost savings in the Pediatric Emergency Department have been achieved by enhancing sedation and analgesia procedures (8).

These examples highlight the potential of simulation projects to generate substantial economic benefits. However, it remains critical to implement research on return on investment and cost-benefit analysis in simulation (36). Connecting the perspectives of clinical educators and managers/policy-makers can be facilitated by Public Health specialists, providing an opportunity for reciprocal exchange of needs and expertise to maximize efforts in QI and PS.

### Training on non-technical skills and human factors

Non-Technical Skills (NTS) are defined as cognitive and social skills, exhibited by individuals and teams, needed to reduce error and improve human performance in complex systems (37). These include Leadership, Decision Making, Teamwork, Situational Awareness, and Communication. Not only in clinical settings, but also in Public Health, simulation plays a crucial role in NTS mastery, as shown in a study on leadership skills (38).

The study of Human Factors (or ergonomics) appears to be a key point for Public Health professionals. Ergonomics, according to the International Ergonomics Association, is “the scientific discipline concerned with the understanding of interactions among humans and other elements of a system, and the profession that applies theory, principles, data and methods to design in order to optimize human wellbeing and overall system performance” (39).

According to Hignett et al., it is desirable to promote an integrated approach between ergonomics and QI science to enhance healthcare safety (40).

Public Health professionals are involved in Multi-Team systems (MTS) in response to Public Health emergencies. In this complex context, as experienced during the recent pandemic, it has become clear that relying solely on technical skills is not enough. To ensure the safe and efficient performance of the MTS, the development of NTS, such as leadership, communication and decision-making, is necessary (41).

### Improvement of clinical outcomes

Promising results regarding the improvement of clinical outcomes are well-described in the literature. In particular, teams using participatory simulation are reported to have improved survival rates for in-hospital cardiac arrest patients (42). Moreover, in the pediatric setting, a simulation program was able to significantly improve the survival rates of pediatric patients undergoing cardiopulmonary arrest (43).

### Patient and caregiver engagement

The topic of patient engagement in the care process is widely covered. The WHO also believes that the involvement of patients and families is a fundamental strategy for promoting safety in healthcare (44).

WHO also highlights the enormous benefits achieved through patient involvement, which would lead to a potential reduction in the huge burden of avoidable patient harm, by up to 15%, saving many lives and costs. Consequently, patient and family involvement has been incorporated as a core principle in the WHO Global Patient Safety Action Plan 2021–2030 (14).

Simulation can play a crucial and innovative role in engaging patients and caregivers. Kneebone et al. (45) advocate the use of simulation to involve patients and physicians in seeking and sharing solutions to healthcare problems through the exchange of different perspectives. Various experiences described in the literature have led not only to greater patient and caregiver safety in the home management of illnesses but also to a reduction in hospital readmissions, with consequent notable cost savings (46).

### Innovation and research

A recent study analyzes the capabilities of ChatGPT, an artificial intelligence-based program, to support simulationists in scenario development processes (47). A simulation-based randomized clinical trial tested the use of a tablet application as a cognitive aid to improve pediatric cardiac arrest management (48). The research highlights how simulation can facilitate the integration of innovative tools into clinical practice, pursuing quality and safety for patients, and how simulation itself can be influenced by the contribution of artificial intelligence. Research and innovation find in simulation a strong orientation toward the multidisciplinary and multi-professional approach. This fosters synergistic interaction between different perspectives, which provides inspiration for innovative solutions in healthcare.

### Ethics and safety

The traditional “see one—do one—teach one” methodological approach, reflecting a training culture embedded especially in the surgical field, is counterbalanced by the motto “never the first time on the patient,” which sums up the increasing focus on PS (49, 50). Whenever possible, it is unethical, because it is unsafe, not to take advantage of simulation, as it provides an ethically unique educational opportunity by ensuring that students practice, through various modalities, before interacting with real patients (51).

The simulation environment offers students biological and psychological safety. Ensuring a safe learning environment is crucial, and this involves actively addressing defensive mechanisms that could hinder the learning process, adhering to the fundamental principles of psychological safety (52).

Furthermore, issues of social justice and equity can be addressed through tailored simulations or by integrating aspects related to implicit bias and social determinants of health into simulation programs (53–55). This aspect could support professionals in maintaining a high ethical standard and a strong sense of professional responsibility in their daily decisions (56) aligned with the UN's 10th Sustainable Development Goal “reduce inequalities” (2) including educational inequalities (15).

We explored various aspects of simulation that can have a significant impact to improve healthcare quality and PS. These considerations show how simulation is an effective experiential tool for knowledge acquisition, skills development, design and test, in different areas of health care, fostering inter-professional collaboration and interdisciplinary approaches.

## Conclusion

In this perspective, we strongly support simulation to enhance QI and PS across diverse professions, starting with Public Health specialists.

Considering WHO's “Ten threats to global health,” addressing these challenges comprehensively is urgent. Simulation is recognized as pivotal for sharing essential knowledge and skills among healthcare professionals (29).

Simulation plays a key role in addressing the universal challenges of healthcare, including educational inequalities. It improves morbidity and mortality rates, enhances patient experiences, optimizes healthcare processes and systems, and strengthens workforce resilience within a culture of safety (15).

Integrating a simulation-based pilot project for Public Health education in our academic environment, including the School of Medicine and its postgraduate programs, aligns with WHO's “Global Patient Safety Action Plan” and responds to the call for action of the “Global Consensus statement on simulation-based practice in healthcare” (14, 15). This initiative fosters collaborative research and enhances training for Public Health professionals. Moreover, active participation in events like World Patient Safety Day can apply academic insights to advance community welfare culturally, socially, and economically.

In conclusion, the PPHSL aims to train professionals capable of contributing to QI and PS. We look forward to further discussion and exploration of whether and how to integrate simulation-based education into the broader Public Health framework, seeing it as an unmissable opportunity to foster interdisciplinary collaboration, promote innovation in health education, and ultimately improve quality and patient care.

## Data availability statement

The original contributions presented in the study are included in the article/supplementary material, further inquiries can be directed to the corresponding authors.

## Author contributions

TB: Conceptualization, Writing – original draft, Writing – review & editing, Methodology, Supervision, Investigation, Project administration. FB: Methodology, Writing – review & editing, Conceptualization, Writing – original draft, Investigation. CB: Supervision, Writing – review & editing, Investigation. AB: Supervision, Writing – review & editing, Investigation. SC: Supervision, Writing – review & editing, Investigation. MF: Supervision, Writing – review & editing, Investigation. VB: Project administration, Supervision, Writing – review & editing, Methodology, Conceptualization, Visualization.
